# Trends in incidence, survival and initial treatments of gynecological sarcoma: a retrospective analysis of the United States subpopulation

**DOI:** 10.1186/s12905-023-02161-1

**Published:** 2023-01-09

**Authors:** Xi He, Qiang Dong, Changfang Weng, Jianfen Gu, Qiao Yang, Guangrong Yang

**Affiliations:** 1 Department of Obstetrics and Gynecology, The People’s Hospital of Sanjiang Dong Autonomous County, Liuzhou, 545500 China; 2 Department of General Medicine, The People’s Hospital of Qijiang District, Chongqing, 401420 China; 3 Department of Oncology, The People’s Hospital of Qijiang District, Chongqing, 401420 China; 4 Department of Clinical Nutrition, The People’s Hospital of Qijiang District, Chongqing, 401420 China; 5 Department of Ultrasound, The 941St Hospital of the PLA Joint Logistic Support Force, Xining, 810007 China

**Keywords:** Gynecologic sarcoma, Epidemiology, Survival, Treatment, SEER

## Abstract

**Background:**

To estimate the incidence, prevalence and incidence-based mortality in patients with gynecologic sarcoma (GS), and described the trends of survival and initial treatments in the US by using the Surveillance, Epidemiology, and End Results (SEER) database.

**Methods:**

GS cases aged 20 years or older between 1975 and 2015 were identified from SEER 9 registries. Incidence, prevalence, and incidence-based mortality were estimated, all rates were age adjusted to the 2000 US standard population and presented as per 100,000 persons. Annual percentage change (APC) and average APC (AAPC) were calculated to describe the trends. In addition, stage distribution, cancer-specific survival (CSS) and initial treatment pattern over time were also reported.

**Results:**

The overall age-adjusted incidence of GS increased from 2.38 to 3.41 per 100,000 persons from 1975 to 2015, with an AAPC of 1.0 (*P* < 0.05), and the AAPC increased to 1.3 (*P* < 0.05) in the last decade. The incidence of population aged ≥ 55 years was three or more times than that of population aged 20–54 year from 1975 to 2015. Corpus and uterus GS was the main subtype, and it increased significantly during last three decades (an APC of 1.5). In addition, the prevalence of corpus and uterus GS increased mostly among all GSs. The incidence of GS with regional and distant stages increased pronouncedly, but not for local stage. GS cases showed increasing 3-year and 5-year CSS rates except for other sites GS. Approximately 87.7% GS cases received surgery during the first-course treatment, but the proportion decreased over years. In contrast, the proportion of receiving multiple treatment modalities increased.

**Conclusions:**

The incidence of GS increased significantly with improved survival, which might due to the strategy of combination of multiple treatment. However, no obvious improvement on the early detection of GS was found, which should be facilitated to further improve the prognosis of GS.

**Supplementary Information:**

The online version contains supplementary material available at 10.1186/s12905-023-02161-1.

## Introduction

Gynecological sarcoma (GS) is a type of rare malignancy, accounting for 13% of all sarcomas and 3–4% of all gynecological malignancies. The most common primary site of GSs is the uterus (83%), followed by the ovaries (8%), vulva and vagina (5%) and other gynecologic organs (2%) [1]. GSs are more aggressive than other gynecological malignancies [2–4]. The common histological types of GSs comprise of carcinosarcoma (malignant mixed mesodermal tumor), leiomyosarcoma, endometrial stromal sarcoma, adenosarcoma and undifferentiated sarcoma [5–10].

Given the low incidence and histological diversity, few studies have focused on GSs. Though a few studies reported the epidemiology of GSs with limited histological subtypes [8, 9], or among pediatric population [11, 12], there is no comprehensive study focused on the epidemiology of adult GSs in the US. In addition, the trends of survival and first-course treatments for GSs have not been adequately addressed.

This cross-sectional epidemiological analysis aimed to estimate the incidence, prevalence and incidence-based mortality in adult patients with GSs, and described the trends of survival and initial treatments in the US by using the Surveillance, Epidemiology, and End Results (SEER) 9 registries, which include approximately 10% of the US population.

## Methods

### Study population

In this study, data was extracted from the SEER 9 registries (SEER-9: Connecticut, Iowa, New Mexico, Utah, Hawaii, Detroit, San Francisco-Oakland, Atlanta, and Seattle-Puget Sound; SEER*Stat software, version 8.3.9; https://seer.cancer.gov/seerstat/). The following criteria were used to identify eligible GS cases: female, aged 20 years or older, one primary only, histologically confirmed, not reported by autopsy or death certificate only, diagnosed between 1975 and 2015. The primary tumor sites were divided into four categories, cervix uteri (C530-C539), corpus and uterus (C540-C549, C559), ovary (C569), other sites (C510-C519, C520, C570-C579, C589). The histological types of GSs comprise of sarcoma (8800–8941/3, 8963/3, 8982/3, 8983/3, 8990/3, 8991/3) and carcinosarcoma (8950/3, 8951/3, 8980/3, 8981/3), as per the *International Classification of Diseases for Oncology, Third Edition (ICD-O-3) *[13].

### Study designs

Incidence, prevalence, and incidence-based mortality were estimated by the SEER*Stat software. All rates were age adjusted to the 2000 US standard population and presented as per 100,000 persons. The trends or the change in incidence and incidence-based mortality were evaluated by annual percentage change (APC) and average APC (AAPC), which were calculated by the NCI’s Joinpoint Regression Program (version 4.9.0.0; https://surveillance.cancer.gov/joinpoint/).

Cancer-specific survival (CSS) was defined as the time from the diagnosis of GSs to death by cancer. The 3-year and 5-year CSS rates, as well as the changes in rates over years, were summarized. First-course treatments, including surgery, chemotherapy, and/or radiation, were also summarized for the overall population and by primary sites, as well as the changes in different periods. In addition, the peaking age period of GSs incidence and the SEER stage distribution were also summarized. The SEER stage was derived from Collaborative Stage for 2004–2015 and Extent of Disease from 1973–2003. The SEER stage comprises of a simplified version of stage: in situ, localized, regional, distant, and unknown. In this study, the variable of in situ was excluded. All these data were calculated by the SEER*Stat software.

### Statistical analysis

Kaplan–Meier method was used to estimate the CSS rate. The *t-*test was used to calculate APC and AAPC, and the values were compared to zero. A 2-tailed estimate of α = 0.05 indicated statistical significance.

## Results

### Trends in incidence

**Fig. 1:**
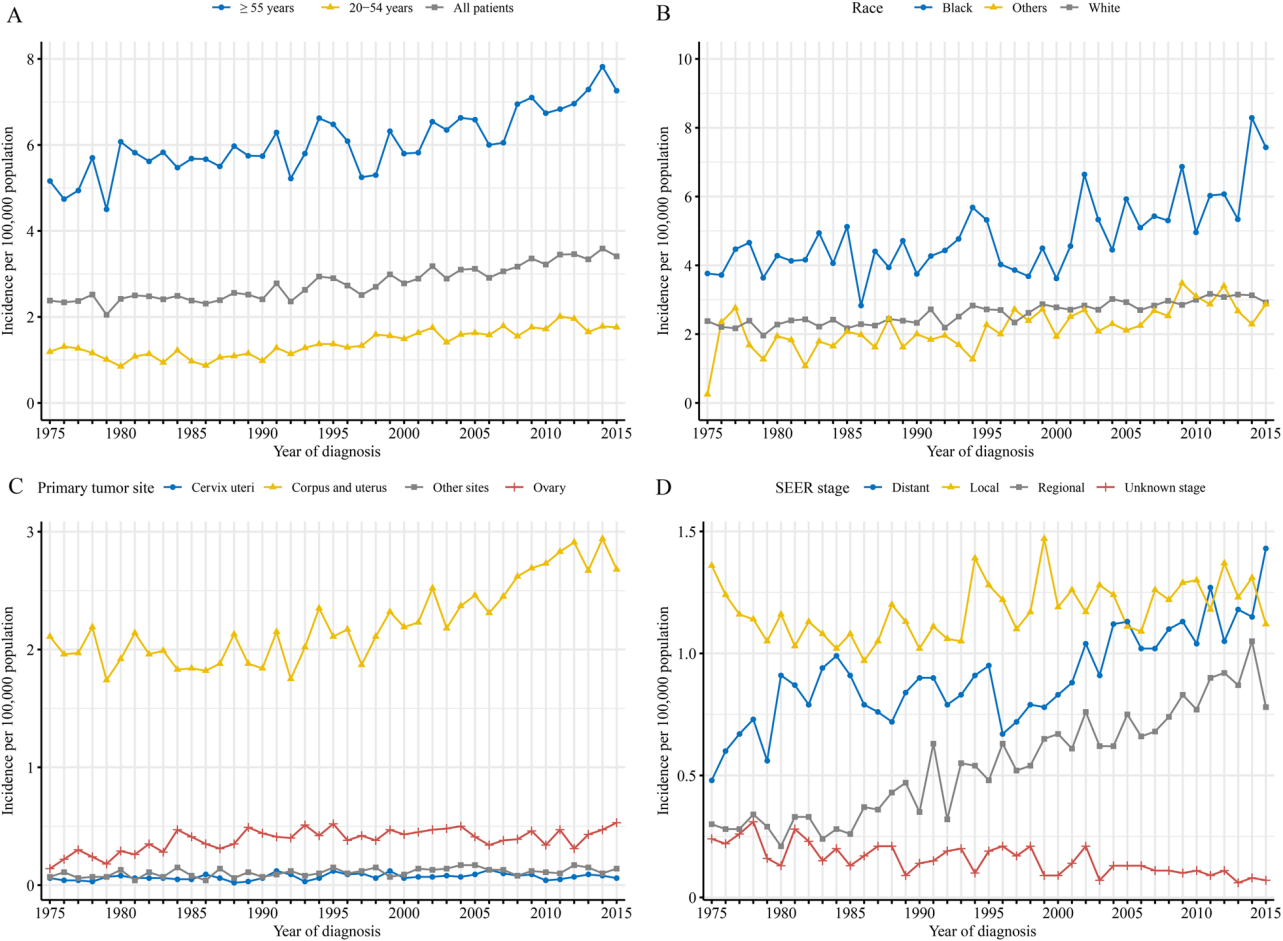
Incidence of gynecologic sarcoma. **A** Overall and by age, **B** by race, **C** by primary tumor sites, **D** by Surveillance, Epidemiology, and End Results stage

The overall age-adjusted incidence of GSs was 2.38 per 100,000 persons in 1975, which increased to 3.41 per 100,000 persons by 2015, with an AAPC of 1.0 (*P* < 0.05), and the AAPC increased to 1.3 (*P* < 0.05) in the last decade (2006–2015) (Fig. 1A and Additional file 1: Table S1). The incidences of GS in patients aged 20–54 years and aged ≥ 55 years increased significantly. The overall AAPC for patients aged 20–54 years and in the last decade was 1.0 and 2.0 (*P* < 0.05), respectively, and that was 0.9 and 1.9 (*P* < 0.05), respectively, for patients aged ≥ 55 years (Fig. 1A and Additional file 1: Table S1). Black population had relatively higher incidence and increased trend compared to White population and other race populations (Fig. 1B and Additional file 1: Table S1). Analysis of different primary sites revealed that the incidence of corpus and uterus GSs increased from 2.11 to 2.68 per 100,000 persons from 1975 to 2015, with an AAPC of 0.8 for 1975–2015 (*P* < 0.05) and 1.5 for 2006–2015 (*P* < 0.05). The incidences of ovary GSs (0.14 to 0.53 per 100,000 persons; AAPC 2.1, *P* < 0.05) and other sites GSs (0.07 to 0.14 per 100,000 persons; AAPC 1.2, *P* < 0.05) also showed increasing trends. In contrast, the incidence of cervix uteri GSs showed no obvious change (0.06 to 0.06 per 100,000 persons; AAPC 0.9, *P* > 0.05) (Fig. 1C and Additional file 1: Table S1). Notably, the increasing trend of ovary GS disappeared in the last decade (an AAPC of 0.2 for year 2006–2015, *P* > *0.05*). The incidence of GS with different stages also showed increasing trends, which was more pronounced in patients with regional stage. While the incidence of GSs with unknown stage showed decreased trend (AAPC-2.7, *P* > 0.05) (Fig. 1D and Additional file 1: Table S1).

Furthermore, analyses of trends by primary sites and stages were performed. As shown in Additional file 2: Fig. S1 and Additional file 3: Table S2, the incidence increased significantly in GSs located in corpus and uterus, ovary and other sites with regional and distant stages. The details of APCs in different time periods are summarized in Additional file 1: Table S1 and Additional file 3: Table S2.

The incidences of GS located in corpus and uterus, and ovary increased with age, peaked at 70–74 years, and subsequently decreased. However, the incidences of GSs located in cervix uteri and other sites did not change significantly with age (Additional file 4: Fig. S2).

### Prevalence and incidence-based mortality

**Fig. 2:**
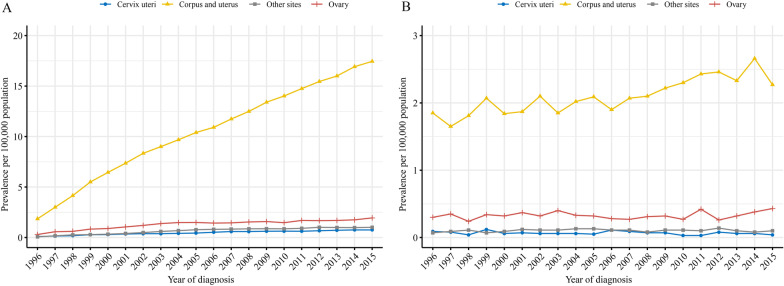
Prevalence of gynecologic sarcoma. **A** 20-year limited-duration prevalence by primary tumor sites, **B** annual prevalence by primary tumor sites

As shown in Fig. 2A, the 20-year limited-duration prevalence of corpus and uterus GS increased significantly, from 1.85 to 17.45 per 100,000 persons from 1996 to 2015. However, the 20-year limited-duration prevalence of cervix uteri, ovary and other sites GS increased slowly. In addition, an increase in annual prevalence of corpus and uterus GS was also observed, from 1.85 to 2.27 per 100,000 persons from 1996 to 2015. The annual prevalence of cervix uteri, ovary and other sites GS showed no obvious changes (Fig. 2B).

In the last decade, the incidence-based mortality for corpus and uterus GS (AAPC 1.2, *P* < *0.05*) and other sites GS (AAPC 3.3, *P* < *0.05*) showed increasing trends. The trends of incidence-based mortality for cervix uteri and ovary GS demonstrated no significant change in the last decade (Additional file 5: Fig. S3 and Additional file 6: Table S3).

### Stage distribution

**Fig. 3:**
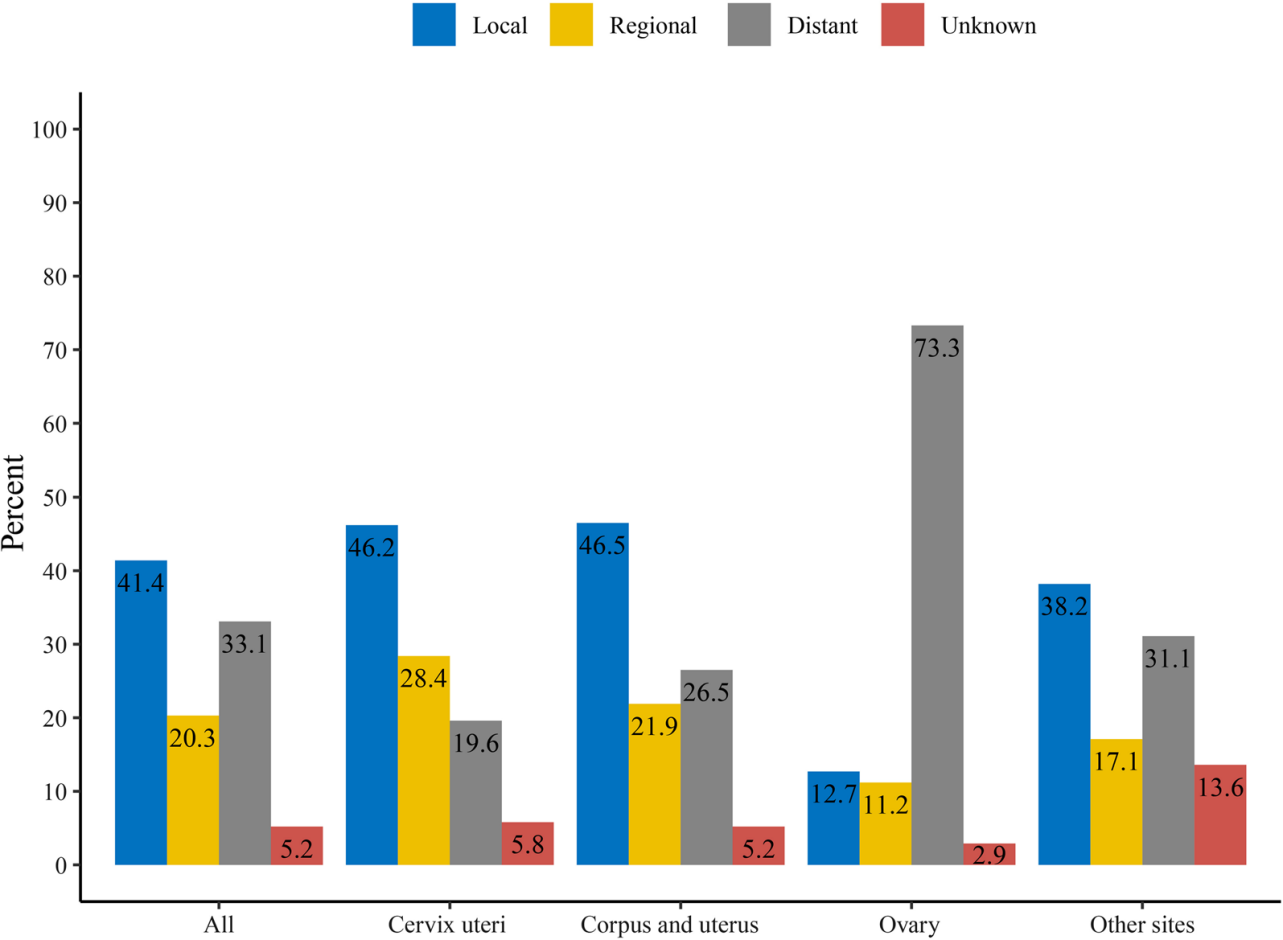
Stage distribution of gynecologic sarcoma

For all GSs cases, local stage accounted for 41.4%, followed by distant stage (33.1%), and regional stage (20.3%). Approximately 5.2% of GS cases had unknown stage. GS with different primary tumor sites had different stage distribution. GS located in cervix uteri, corpus and uterus, and other sites presented with higher proportions of local stage. In contrast to that, the most common stage for ovary GS was distant stage, up to 73.3% (Fig. 3).

### Cancer-specific survival

**Fig. 4:**
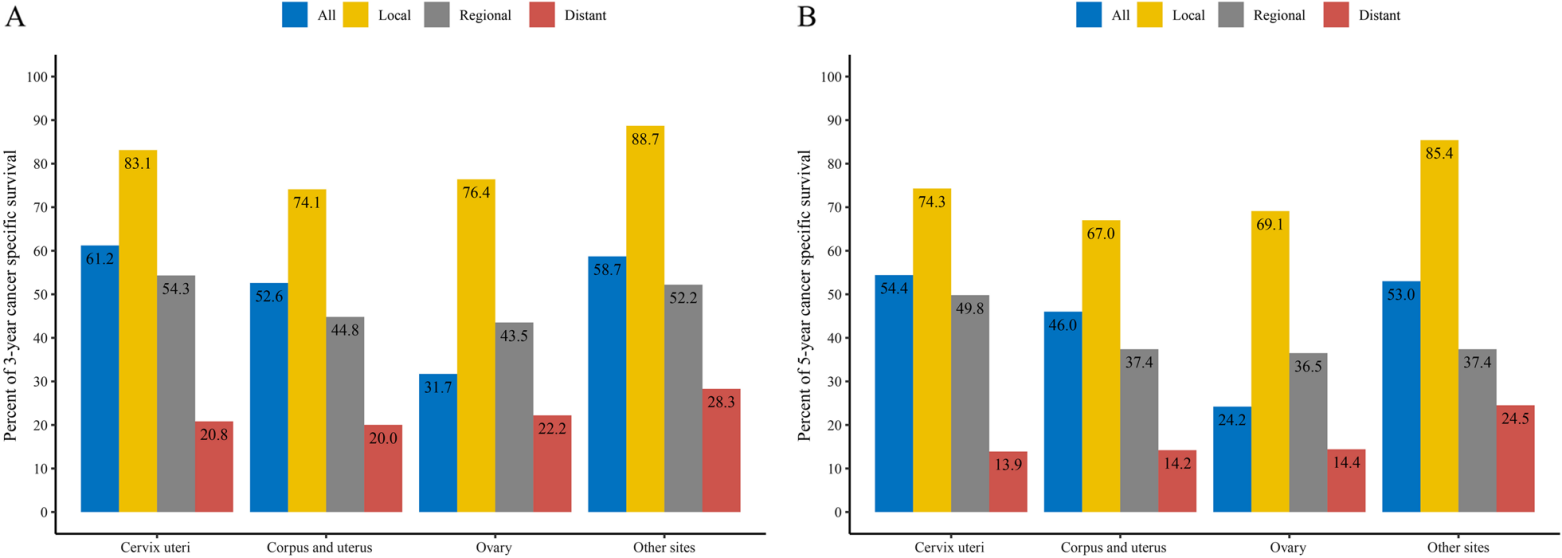
Cancer-specific survival rate of gynecologic sarcoma. **A** 3-year cancer-specific survival rate by primary tumor sites, **B** 5-year cancer-specific survival rate by primary tumor sites

Overall, the estimated 3-year CSS rate for cervix uteri, corpus and uterus, ovary and other sites GS was 61.2%, 52.6%, 31.7% and 58.7%, respectively. Moreover, the estimated 5-year CSS rate was 54.4%, 46.0%, 24.2% and 53.0%, respectively. For cases with local and distant stages, other sites GS presented with better 3-year and 5-year CSS rates compared to other GSs. For cases with regional stage, cervix uteri GS presented the best 3-year CSS and 5-year CSS rates. The details of 3-year and 5-year CSS rates are presented in Fig. 4.

**Fig. 5:**
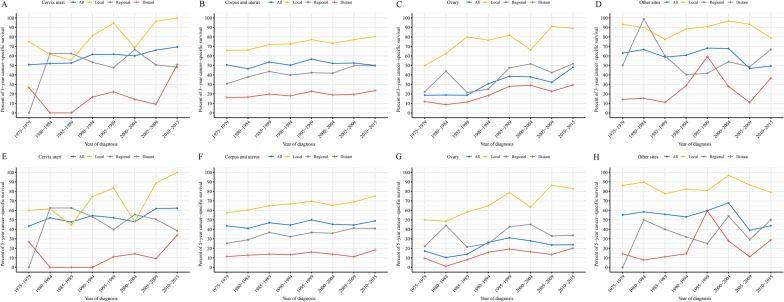
Trends in cancer-specific survival rate. **A**–**D** trends in 3-year cancer-specific survival rate by primary tumor sites, **E**–**H** trends in 5-year cancer-specific survival rate by primary tumor sites

From 1975 to 2015, cervix uteri and ovary GS had improved 3-year and 5-year CSS rates, and the improvements were more pronounced in local stage (Fig. 5A, C, E, G). Though the overall 3-year and 5-year CSS rates of corpus and uterus GS had no obvious improvement from 1975 to 2015, the rates of all cases with known stages all increased significantly (Fig. 5B, F). The 3-year and 5-year CSS rates of other sites GS decreased, mainly due to the decreases in local stage (Fig. 5D, H).

### Initial treatment and trends

Overall, approximately 87.7% of GS cases received surgery, and approximately 39.9% cases received surgery as the only treatment modality during the first-course treatment. The proportions of cases receiving chemotherapy and radiation were 38.2% and 25.7%. Treatment pattern for each primary site was generally consistent with the overall population, except for a much higher proportion of chemotherapy and a lower proportion of radiation in ovary GS cases. Overall, approximately 6.4% of cases received no treatment, and it was more pronounced in cervix uteri GSs cases (11.8%). The details of treatment patterns are summarized in Table 1.

**Table1:** Initial treatment and trends for gynecologic sarcoma

	*N*	No treatment	Surgery	Chemotherapy	Radiation	Single treatment				Multiple treatment
						Only one treatment	Surgery only	Chemotherapy only	Radiation only	
All patients	23018	1469 (6.4%)	20183 (87.7%)	8788 (38.2%)	5923 (25.7%)	10299 (44.7%)	9193 (39.9%)	645 (2.8%)	461 (2.0%)	11250 (48.9%)
*Cervix uteri*	617	73 (11.8%)	439 (71.2%)	176 (28.5%)	240 (38.9%)	296 (48.0%)	227 (36.8%)	15 (2.4%)	54 (8.8%)	248 (40.2%)
1975–1984	42	6 (14.3%)	31 (73.8%)	2 (4.8%)	18 (42.9%)	21 (50%)	16 (38.1%)	1 (2.4%)	4 (9.5%)	15 (35.7%)
1985–1994	66	7 (10.6%)	43 (65.2%)	18 (27.3%)	33 (50.0%)	31 (47.0%)	19 (28.8%)	0 (0%)	12 (18.2%)	28 (42.4%)
1995–2004	186	20 (10.8%)	139 (74.7%)	38 (20.4%)	71 (38.2%)	99 (53.2%)	81 (43.5%)	3 (1.6%)	15 (8.1%)	67 (36.0%)
2005–2015	323	40 (12.4%)	226 (70.0%)	118 (36.5%)	118 (36.5%)	145 (44.9%)	111 (34.4%)	11 (3.4%)	23 (7.1%)	138 (42.7%)
*Corpus and uterus*	18422	1144 (6.2%)	16267 (88.3%)	6312 (34.3%)	5352 (29.1%)	8514 (46.2%)	7703 (41.8%)	430 (2.3%)	381 (2.1%)	8764 (47.6%)
1975–1984	1524	102 (6.7%)	1290 (84.6%)	284 (18.6%)	565 (37.1%)	781 (51.2%)	676 (44.4%)	23 (1.5%)	82 (5.4%)	641 (42.1%)
1985–1994	1961	105 (5.4%)	1743 (88.9%)	444 (22.6%)	599 (30.5%)	1018 (51.9%)	922 (47.0%)	34 (1.7%)	62 (3.2%)	838 (42.7%)
1995–2004	4897	310 (6.3%)	4383 (89.5%)	1153 (23.5%)	1445 (29.5%)	2500 (51.1%)	2334 (47.7%)	75 (1.5%)	91 (1.9%)	2087 (42.6%)
2005–2015	10,040	627 (6.2%)	8851 (88.2%)	4431 (44.1%)	2743 (27.3%)	4215 (42.0%)	3771 (37.6%)	298 (3.0%)	146 (1.5%)	5198 (51.8%)
*Ovary*	3133	174 (5.6%)	2769 (88.4%)	2023 (64.6%)	154 (4.9%)	1068 (34.1%)	887 (28.3%)	174 (5.6%)	7 (0.2%)	1891 (60.4%)
1975–1984	214	11 (5.1%)	185 (86.4%)	116 (54.2%)	34 (15.9%)	85 (39.7%)	69 (32.2%)	13 (6.1%)	3 (1.4%)	118 (55.1%)
1985–1994	408	13 (3.2%)	384 (94.1%)	254 (62.3%)	19 (4.7%)	145 (35.5%)	135 (33.1%)	10 (2.5%)	0 (0%)	250 (61.3%)
1995–2004	911	42 (4.6%)	834 (91.5%)	535 (58.7%)	47 (5.2%)	353 (38.7%)	321 (35.2%)	32 (3.5%)	0 (0%)	516 (56.6%)
2005–2015	1600	108 (6.8%)	1366 (85.4%)	1118 (69.9%)	54 (3.4%)	485 (30.3%)	362 (22.6%)	119 (7.4%)	4 (0.3%)	1007 (62.9%)
*Other sites*	846	78 (9.2%)	708 (83.7%)	277 (32.7%)	177 (20.9%)	421 (49.8%)	376 (44.4%)	26 (3.1%)	19 (2.2%)	347 (41.0%)
1975–1984	68	3 (4.4%)	60 (88.2%)	16 (23.5%)	11 (16.2%)	45 (66.2%)	42 (61.8%)	2 (2.9%)	1 (1.5%)	20 (29.4%)
1985–1994	98	4 (4.1%)	88 (89.8%)	26 (26.5%)	23 (23.5%)	55 (56.1%)	50 (51.0%)	3 (3.1%)	2 (2.0%)	39 (39.8%)
1995–2004	268	20 (7.5%)	228 (85.1%)	73 (27.2%)	67 (25.0%)	146 (54.5%)	130 (48.5%)	5 (1.9%)	11 (4.1%)	103 (38.4%)
2005–2015	412	51 (12.4%)	332 (80.6%)	162 (39.3%)	76 (18.4%)	176 (42.7%)	154 (37.4%)	16 (3.9%)	6 (1.5%)	185 (44.9%)

The changes in treatment pattern for each primary site from 1975 to 2015 are also described in Table 1, and notable shifts were observed. In general, the proportions of cases receiving surgery and radiation decreased, except for the proportion receiving surgery increased in corpus and uterus GS cases. The proportion receiving chemotherapy increased over the years. Moreover, the proportion receiving only one treatment modality as first-course treatment decreased, mainly due to the decrease in surgery only. In contrast, the proportion receiving two or more treatment modalities increased significantly.

## Discussion

To the best of our knowledge, this is the first US subpopulation-based study, using a large amount of data integrated in the SEER program, to perform a comprehensive analysis of GS cases in the US from 1975 to 2015, with a focus on epidemiology, survival and initial treatment. Some notable findings are discussed below.

In this study, we found that the incidence of GS gradually increased by 1.0% each year, but it increased to 1.3% each year in the last decade. This increase may be partly related to radiation, exogenous estrogen and obesity [14, 15]. The changes in trends in both age subgroups were similar, while the incidence in the population aged ≥ 55 years was three or more times than that of the population aged 20–54 years from 1975 to 2015. In addition, the incidence of all GSs peaked at a relatively older age period. Notably, the incidence and increasing trend were more pronounced in Black population. This was consistent with the results of a previous study on the epidemiological analysis of uterine sarcomas in the US [8]. A probable explanation could be that obesity was higher among Black women in the US, which was a risk factor for GS occurrence [14, 15].

Corpus and uterus GS was the main subtype of all GSs in this study, and it increased significantly during the last three decades (an APC of 1.5 for 1986–2015). In addition, the 20-year limited-duration prevalence and the annual prevalence of corpus and uterus GS also increased. The probable reason could be that the most common histological subtype of GS was carcinosarcoma, accounting for approximately 50% [5], which had genetic predisposition in the corpus and uterus [16]. For the past four decades, ovary GS had the highest increasing trend, followed by other sites GS.

GSs with different SEER stages presented with different trends of incidence. GSs with regional and distant stages increased significantly, while GSs with unknown stage decreased significantly. The evolution and improvement of diagnostic techniques, such as computed tomography (CT), magnetic resonance imaging, positron emission tomography-CT, could help to better characterize the stage at diagnosis [17, 18]. Notably, the increase in incidence of local GSs was not obvious.

Previous studies demonstrated that the survival of ovary GS was inferior to uterine GS [19, 20]. In this study, we also found better 3-year and 5-year CSS rates in corpus and uterus GS compared to ovary GS. A significantly higher proportion of distant stage of ovary GS at diagnosis (73.3%) and the low 3-year (22.2%) and 5-year (14.4%) CSS rates for ovary GS at distant stage contributed to the worst survival. Most patients with corpus and uterus GS or other sites GS had abnormal vaginal bleeding, which facilitated early diagnosis and timely treatment. In contrast, patients with ovary GSs presented with non-specific symptoms, which led to delay in diagnosis [10, 21]. Besides, the higher proportions of local and regional stages in cervix uteri GS cases, as well as relatively better survival for cervix uteri GS cases with local and regional stages, contributed to the best 3-year and 5-year CSS rates for overall cervix uteri GS cases.

In this study, the main treatment modality for GS was surgery, which was consistent with previous reports [10, 22]. In addition, individualized, diversified and synthesized treatments based on the heterogeneity of each GS case are warranted [7, 23–25]. Moreover, targeted therapy and immunotherapy were considered as novel and promising treatment modalities for GSs [1, 26–28]. There is no standard treatment regime for some rare GSs, such as ovary GS [10, 21]. Some studies suggested the treatment strategy of ovary GS should be consistent with that of epithelial ovarian cancer [4], while other studies recommended that the treatment for ovary GSs should follow the principle of treatment for corpus and uterus GS [29]. Further studies are warranted to better explore the standard treatment for rare GSs.

This study firstly summarized the trends of treatment patterns of GSs over the years in the US. We found that the proportions of surgery and single treatment decreased over the years. This might be due to the increasing incidences of GSs with regional and distant stages. Instead, the proportion of multiple treatments increased significantly. The increased availability of chemotherapy and radiation treatment data might be related to improved access and reporting mechanisms for treatment information provided outside the hospital setting. These may explain the improvements of 3-year and 5-year CSS rates of cervix uteri, corpus and uterus, and ovary GSs. The proportions of untreated GSs in ovary and other sites increased over years. and the proportion increased by approximately three times for other sites GSs. This may result in the decrease in 3-year and 5-year CSS rates of the other sites GS. Notably, part of the GS cases categorized as receiving no treatment may be misclassified, because the SEER database did not include first-line targeted therapy or immunotherapy.

GSs presented with worse outcomes compared to other gynecological malignancies [9]. A study reported that patients with cervical sarcoma had the worst 5-year overall survival rate among all cervical cancer cases (63.3% for squamous cell carcinoma, 73.7% for adenocarcinoma, and 47.7% for sarcoma, *P* < 0.001), and patients with cervical sarcoma had a higher risk of death compared to other patients [2]. A study found that patients with uterine carcinosarcoma presented with higher tumor grades, a higher proportion of metastatic disease and worse survival than patients with endometrioid adenocarcinoma [3]. A study demonstrated a dismal survival for ovarian sarcomas compared to epithelial ovarian carcinomas when matched with the same International Federation of Gynecology and Obstetrics (FIGO) stage [4]. A study suggested that vestibular gland sarcoma grows faster and is more invasive than squamous cell carcinoma or adenoid cystic carcinoma [30]. All the above-mentioned studies indicated that GSs were more aggressive with poorest survival among all gynecological malignancies.

This study had several limitations. First, the pathological subtypes of GSs are varied. However, because of the rare incidence, the epidemiological analysis by pathological subtypes was not performed. Second, as some important clinical features were not included in the SEER database, the prognostic factors were not adequately estimated. Third, the analysis of treatment patterns had potential bias because of the incompleteness of these two variables and undetected reasons for receiving or not receiving radiation/chemotherapy [31]. Fourth, the SEER stage in this study changed over the study period, which may cause a bias in the analysis of stage distribution to some extent.

## Conclusion

This study performed a comprehensive analysis of the epidemiology, survival and initial treatment of GS from 1975 to 2015 in the US, using the subpopulation data from SEER 9 registries. The incidence of GS increased significantly over the years. Corpus and uterus GS accounted for the majority of all GS, and had significantly increased trends in incidence and prevalence trends. In general, the survival of GS improved over the years, partly due to the strategy of combination of multiple treatments. However, no obvious improvement in the early detection of GS was found, which should be facilitated to further improve the prognosis of GS.

## Supplementary Information

**Additional file 1. Table S1** Trends in incidence of gynecologic sarcoma.

**Additional file 2. Figure S1** Incidence of gynecologic sarcoma by primary tumor sites and Surveillance, Epidemiology, and End Results stage. A) cervix uteri by stage, B) corpus and uterus by stage, C) ovary by stage, D) other sites by stage.

**Additional file 3. Table S2** Trends in incidence of gynecologic sarcoma by primary tumor sites and SEER stage.

**Additional file 4.** **Figure S2** Incidence of gynecologic sarcoma at different age period and by different primary tumor sites.

**Additional file 5.** **Figure S3** Incidence-based mortality of gynecologic sarcoma by different primary tumor sites.

**Additional file 6. Table S3** Trends in incidence-based mortality of gynecologic sarcoma by primary tumor sites.

## Data Availability

The datasets used and/or analysed during the current study available from the corresponding author on reasonable request.
