# Supplementary material for: Trends in incidence, survival and initial treatments of gynecological sarcoma: a retrospective analysis of the United States subpopulation
Source: BMC Womens Health. 2023 Jan 9;23:10. doi: 10.1186/s12905-023-02161-1 (PMC9830743; doi:10.1186/s12905-023-02161-1)
Supplement: Supplementary file 1 — Additional file 1. Table S1 Trends in incidence of gynecologic sarcoma. [file 12905_2023_2161_MOESM1_ESM.docx]

Supplementary Table1: Trends in incidence of gynecologic sarcoma.

|  | **Subgroups** | **Trend 1** | |  | **Trend 2** | |  | **Trend 3** | |  | **AAPC** | |
| --- | --- | --- | --- | --- | --- | --- | --- | --- | --- | --- | --- | --- |
|  |  | **Years** | **APC** |  | **Years** | **APC** |  | **Years** | **APC** |  | **1975-2015** | **2006-2015** |
| **All gynecological sarcomas** |  | 1975-1986 | 0.2 |  | 1986-2015 | 1.3* |  |  |  |  | 1.0* | 1.3* |
| **Age (years)** | 20-54 | 1975-1980 | -5.7 |  | 1980-2015 | 2.0* |  |  |  |  | 1.0* | 2.0* |
|  | ≥ 55 | 1975-2006 | 0.6* |  | 2006-2015 | 1.9* |  |  |  |  | 0.9* | 1.9* |
| **Race** | White | 1975-2015 | 0.9* |  |  |  |  |  |  |  | 0.9* | 0.9* |
|  | Black | 1975-2015 | 1.5* |  |  |  |  |  |  |  | 1.5* | 1.5* |
|  | Others | 1975-2015 | 1.5* |  |  |  |  |  |  |  | 1.5* | 1.5* |
| **Primary tumor site** | Cervix uteri | 1975-2015 | 0.9 |  |  |  |  |  |  |  | 0.9 | 0.9 |
|  | Corpus and uterus | 1975-1986 | -1.1 |  | 1986-2015 | 1.5* |  |  |  |  | 0.8* | 1.5* |
|  | Ovary | 1975-1985 | 8.2* |  | 1985-2015 | 0.2 |  |  |  |  | 2.1* | 0.2 |
|  | Other sites | 1975-2015 | 1.2* |  |  |  |  |  |  |  | 1.2* | 1.2* |
| **SEER stage** | Local | 1975-2015 | 0.3* |  |  |  |  |  |  |  | 0.3* | 0.3* |
|  | Regional | 1975-1996 | 3.9* |  | 1996-2015 | 2.8* |  |  |  |  | 3.4* | 2.8* |
|  | Distant | 1975-1981 | 9.2* |  | 1981-1997 | -0.6 |  | 1997-2015 | 2.7* |  | 2.3* | 2.7* |
|  | Unknown | 1975-2002 | -1.7* |  | 2002-2015 | -4.8* |  |  |  |  | -2.7* | -4.8* |

APC, annual percent change; AAPC, average annual percent change; SEER, Surveillance, Epidemiology, and End Results. * indicates statistical significance (P < 0.05).
