# Supplementary figures and images for: Trends in incidence, survival and initial treatments of gynecological sarcoma: a retrospective analysis of the United States subpopulation
Source: BMC Womens Health. 2023 Jan 9;23:10. doi: 10.1186/s12905-023-02161-1 (PMC9830743; doi:10.1186/s12905-023-02161-1)

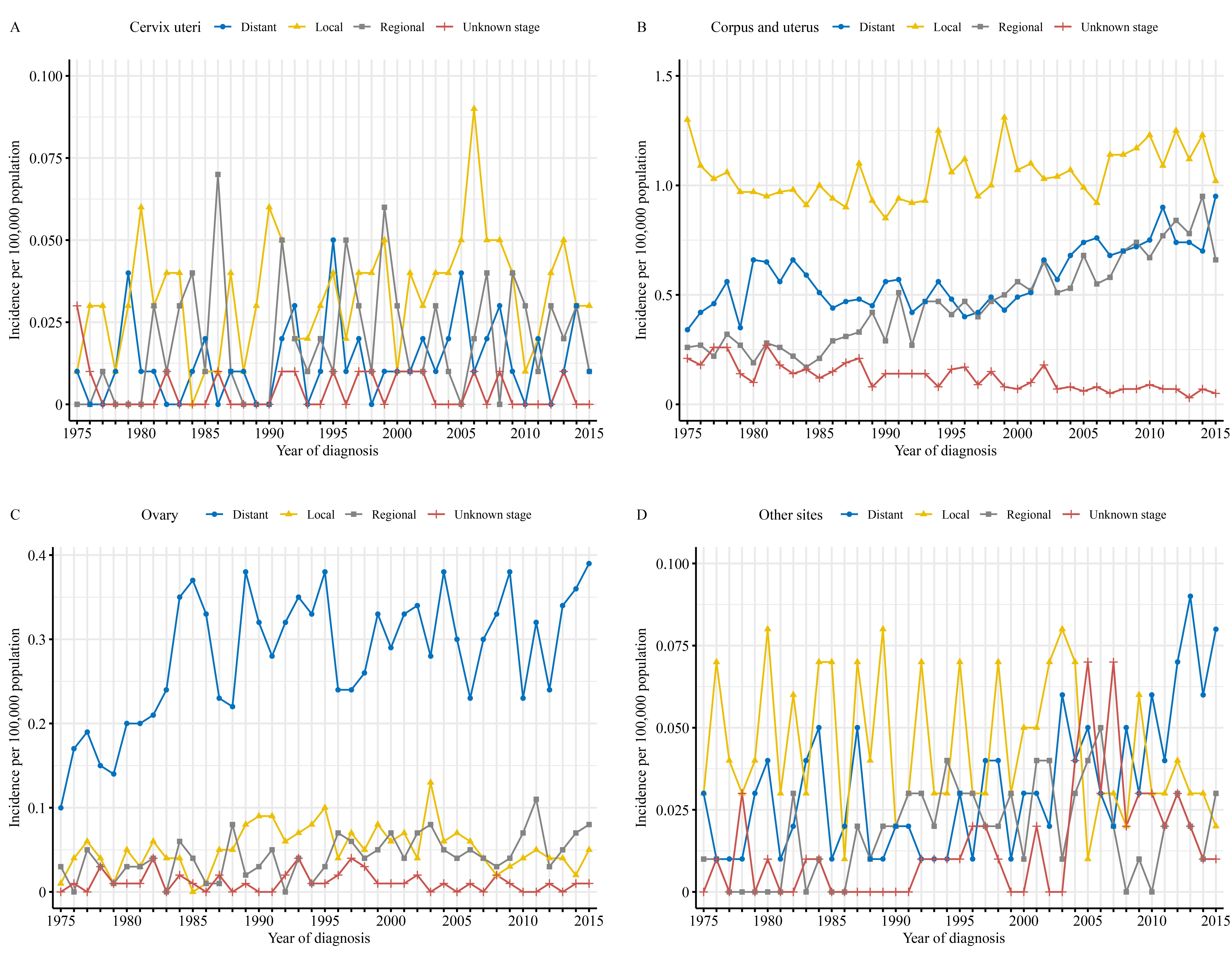

Supplement: Supplementary file 2 — Additional file 2. Figure S1 Incidence of gynecologic sarcoma by primary tumor sites and Surveillance, Epidemiology, and End Results stage. A) cervix uteri by stage, B) corpus and uterus by stage, C) ovary by stage, D) other sites by stage. [file 12905_2023_2161_MOESM2_ESM.jpg]

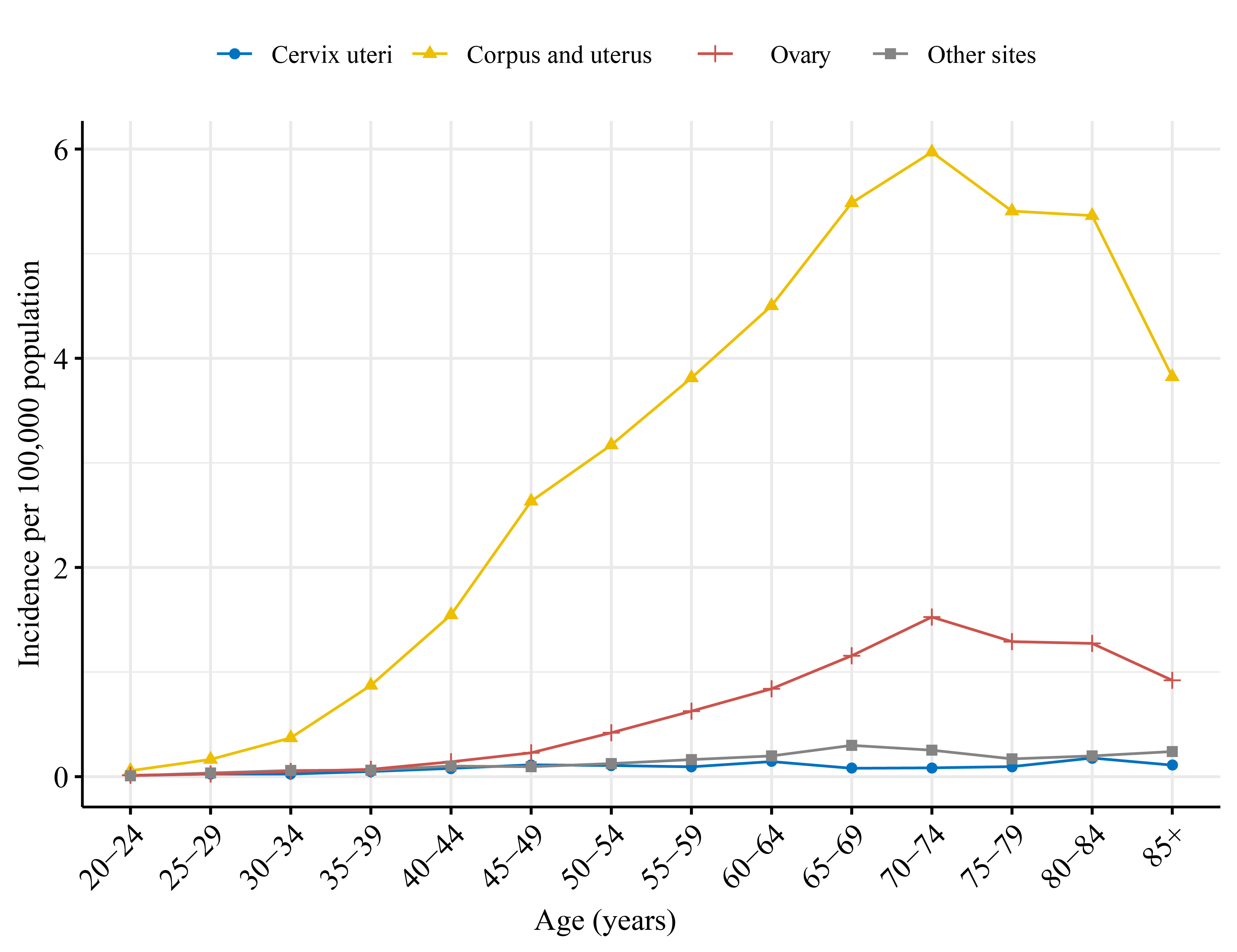

Supplement: Supplementary file 4 — Additional file 4. Figure S2 Incidence of gynecologic sarcoma at different age period and by different primary tumor sites. [file 12905_2023_2161_MOESM4_ESM.jpg]

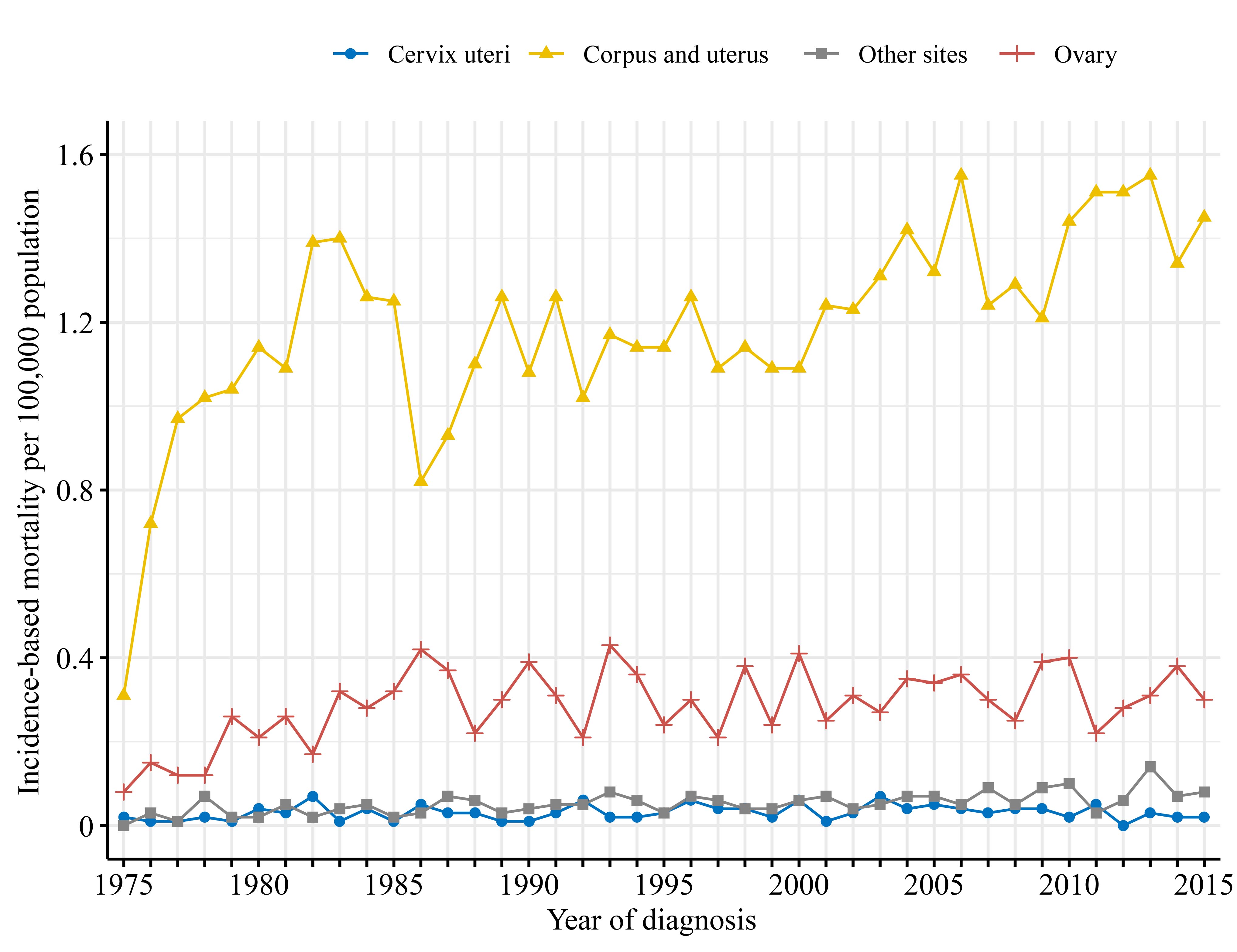

Supplement: Supplementary file 5 — Additional file 5. Figure S3 Incidence-based mortality of gynecologic sarcoma by different primary tumor sites. [file 12905_2023_2161_MOESM5_ESM.jpg]
