# Supplementary material for: Trends in incidence, survival and initial treatments of gynecological sarcoma: a retrospective analysis of the United States subpopulation
Source: BMC Womens Health. 2023 Jan 9;23:10. doi: 10.1186/s12905-023-02161-1 (PMC9830743; doi:10.1186/s12905-023-02161-1)
Supplement: Supplementary file 3 — Additional file 3. Table S2 Trends in incidence of gynecologic sarcoma by primary tumor sites and SEER stage. [file 12905_2023_2161_MOESM3_ESM.docx]

Supplementary Table2: Trends in incidence of gynecologic sarcoma by primary tumor sites and SEER stage.

|  | **Subgroups** | **Trend 1** | |  | **Trend 2** | |  | **Trend 3** | |  | **AAPC** | |
| --- | --- | --- | --- | --- | --- | --- | --- | --- | --- | --- | --- | --- |
|  |  | **Years** | **APC** |  | **Years** | **APC** |  | **Years** | **APC** |  | **1975-2017** | **2008-2017** |
| **Cervix uteri** | Local | 1975-2015 | 1.3 |  |  |  |  |  |  |  | 1.3 | 1.3 |
|  | Regional | 1975-2015 | 5.3 |  |  |  |  |  |  |  | 5.3 | 5.3 |
|  | Distant | 1975-2015 | 4.1 |  |  |  |  |  |  |  | 4.1 | 4.1 |
|  | Unknown | 1975-2015 | -2.2 |  |  |  |  |  |  |  | -2.2 | -2.2 |
| **Corpus and uterus** | Local | 1975-1979 | -6.9 |  | 1979-2015 | 0.6* |  |  |  |  | -0.2 | 0.6* |
|  | Regional | 1975-1984 | -2.8 |  | 1984-1989 | 11.3 |  | 1989-2015 | 3.2* |  | 2.8* | 3.2* |
|  | Distant | 1975-1980 | 10.4 |  | 1980-1996 | -1.6 |  | 1996-2015 | 3.5* |  | 2.3* | 3.5* |
|  | Unknown | 1975-2002 | -2.7* |  | 2002-2015 | -5.4* |  |  |  |  | -3.6* | -5.4* |
| **Ovary** | Local | 1975-2015 | 0.2 |  |  |  |  |  |  |  | 0.2 | 0.2 |
|  | Regional | 1975-2015 | 3.2* |  |  |  |  |  |  |  | 3.2* | 3.2* |
|  | Distant | 1975-1984 | 10.7* |  | 1984-2015 | 0.3 |  |  |  |  | 2.5* | 0.3 |
|  | Unknown | 1975-2015 | -2.4 |  |  |  |  |  |  |  | -2.4 | -2.4 |
| **Other sites** | Local | 1975-2015 | -1.1 |  |  |  |  |  |  |  | -1.1 | -1.1 |
|  | Regional | 1975-2015 | 7.1* |  |  |  |  |  |  |  | 7.1* | 7.1* |
|  | Distant | 1975-2015 | 3.0* |  |  |  |  |  |  |  | 3.0* | 3.0* |
|  | Unknown | 1975-2015 | 13.5* |  |  |  |  |  |  |  | 13.5* | 13.5* |

APC, annual percent change; AAPC, average annual percent change; SEER, Surveillance, Epidemiology, and End Results. * indicates statistical significance (P < 0.05).
