# Supplementary material for: Trends in incidence, survival and initial treatments of gynecological sarcoma: a retrospective analysis of the United States subpopulation
Source: BMC Womens Health. 2023 Jan 9;23:10. doi: 10.1186/s12905-023-02161-1 (PMC9830743; doi:10.1186/s12905-023-02161-1)
Supplement: Supplementary file 6 — Additional file 6. Table S3 Trends in incidence-based mortality of gynecologic sarcoma by primary tumor sites. [file 12905_2023_2161_MOESM6_ESM.docx]

Supplementary Table3: Trends in incidence-based mortality of gynecologic sarcoma by primary tumor sites.

| **Primary site** | **Trend 1** | |  | **Trend 2** | |  | **Trend 3** | |  | **AAPC** | |
| --- | --- | --- | --- | --- | --- | --- | --- | --- | --- | --- | --- |
|  | **Years** | **APC** |  | **Years** | **APC** |  | **Years** | **APC** |  | **1975-2015** | **2006-2015** |
| Cervix uteri | 1975-2015 | -0.3 |  |  |  |  |  |  |  | -0.3 | -0.3 |
| Corpus and uterus | 1975-1983 | 10.7* |  | 1983-1986 | -11.5 |  | 1986-2015 | 1.3* |  | 2.1 | 1.2* |
| Ovary | 1975-1984 | 12.2* |  | 1983-2017 | -0.1 |  |  |  |  | 2.6* | -0.1 |
| Other sites | 1975-2015 | 3.3* |  |  |  |  |  |  |  | 3.3* | 3.3* |

APC, annual percent change; AAPC, average annual percent change. * indicates statistical significance (P < 0.05).
